# Electricity consumption prediction using an advanced spatial-temporal deep learning framework

**DOI:** 10.1038/s41598-026-46825-y

**Published:** 2026-04-01

**Authors:** Vignesh A. Palan, Sumith N.

**Affiliations:** Manipal Institute of Technology, Manipal Academy of Higher Education, Manipal, India

**Keywords:** Electricity forecasting, Deep learning, Time series analysis, Spatial-temporal models, Energy, GRU, Transformer, Energy science and technology, Engineering, Mathematics and computing

## Abstract

Reliable forecasting of electricity usage is vital to support economic growth and economic performance. The inherent non-linearity and complex temporal dependencies in energy demand data pose significant challenges for traditional forecasting methods. This report details an extensive investigation into the application of advanced deep learning (DL) architectures for this task. We propose spatial temporal model, namely Spatial-Temporal GRU (ST-GRU), for predicting the electricity consumption. In this work, the term spatial refers to inter-feature relationships within the multivariate input space rather than geographic spatial information. Utilizing a multi-source 15-minute interval dataset, we employ cyclical feature engineering and a sliding window approach to structure the sequential inputs. We conduct a comparative analysis of a diverse suite of sequence models, including baseline GRU and LSTM networks, alongside more sophisticated architectures such as Bidirectional RNNs, Attention-based models, Temporal Convolutional Networks (TCN), Transformers, and Spatial-Temporal models. Our empirical evaluation on a reserved test set reveals that specialized architectures, particularly the Spatial-Temporal Gated Recurrent Unit (ST-GRU), deliver superior predictive accuracy. The proposed ST-GRU reduced RMSE by 6.4% compared to standard GRU and by approximately 25% compared to LSTM on Dataset 1, while maintaining consistent generalization performance across cross-dataset evaluation. The ST-GRU model demonstrates a notable ability to effectively model the inter-feature relationships (spatial dynamics) before capturing their evolution over time (temporal dynamics), resulting in the lowest prediction error. This outcome highlights the substantial potential of architecturally specialized DL frameworks to advance the precision of energy.

## Introduction

The ability to accurately anticipate electricity consumption is foundational for effective energy grid operations, strategic resource deployment, and sound economic planning. Stakeholders, from industrial consumers to utility providers, depend on reliable forecasts to streamline activities, manage expenditures, and maintain the integrity of the electrical grid. Electricity demand is governed by a complex interplay of multiple factors, including cyclical temporal patterns (hourly, daily, weekly, and annual variations) as well as unexpected events and structural shifts. This multifaceted behavior makes accurate forecasting inherently challenging, often surpassing the capabilities of simpler models that struggle to adequately capture non-linear dependencies and long-range temporal dynamics. In this regard, the Power System Operation Corporation Limited (POSOCO)^1^ provides valuable insights into the evolving energy consumption patterns in India.

Beyond temporal periodicity, electricity demand is also shaped by intricate interdependencies among explanatory variables such as historical load values, calendar indicators, and engineered cyclical features. These interactions are not static; they evolve dynamically over time and significantly influence short-term consumption behavior. Consequently, modeling inter-feature relationships prior to temporal evolution facilitates structured representation learning that purely sequential models may fail to capture, thereby underscoring the importance of architectures capable of integrating both temporal dynamics and feature-level interactions.

Recent advancement made in Artificial Intelligence specifically Deep learning, has spurred the development of more advanced techniques suitable for complex forecasting tasks. To solve this challenge, we propose a novel forecasting framework built upon a comparative evaluation of sophisticated deep learning architectures. This research extends beyond a foundational ML and DL comparison to analyze the performance nuances within a diverse set of models, including Bidirectional RNNs, Attention-based RNNs, Transformers, TCNs, and Spatial-Temporal networks. In this work, the term spatial refers to inter-feature relationships within the multivariate input space rather than geographic spatial information. These are benchmarked against foundational sequence models like GRU and LSTM. By training these distinct model types on high-resolution energy usage data enhanced with temporal features, this study evaluates their forecasting performance to illuminate the relative strengths and potential benefits of specialized deep learning architectures for energy prediction. The main contributions of this study are summarized as follows:

1. Hierarchical spatio-temporal modeling framework: We propose a hierarchical spatio-temporal deep learning architecture that explicitly decouples feature-wise (spatial) dependency modeling from temporal dependency learning, enabling structured representation of multivariate electricity consumption data.
2. Spatial GRU for inter-feature dependency learning: A spatial GRU module is employed to model inter-feature correlations at each time step, transforming high-dimensional concurrent feature vectors into compact latent embeddings that preserve essential spatial dependencies.
3. Temporal GRU for sequential load dynamics: The spatial embeddings are processed by a temporal GRU to capture long-term temporal patterns and historical dependencies in electricity demand, improving forecasting accuracy.
4. Cross-dataset generalization evaluation: The proposed model is trained on one dataset and evaluated on an independent dataset using fixed preprocessing parameters, providing a realistic assessment of cross-dataset generalization under deployment-oriented conditions.

## Background study

The domain of time series forecasting for electricity consumption has been extensively studied, leading to the application of numerous statistical and machine learning strategies. Various research efforts have examined different models and approaches to enhance the precision and utility of forecasts and we present a few here. The reviewed works are grouped based on the primary mechanism used to model spatial and temporal dependencies, specifically whether these dependencies are captured through hybrid physics–data-driven formulations, convolutional feature extractors, attention mechanisms, or graph-based representations. This categorization reflects fundamental architectural differences in how spatial relationships are encoded and fused with temporal dynamics, rather than differences in application domain.

### Hybrid decomposition–optimization models

Pierre et al.^2^ investigate peak electrical energy consumption prediction, comparing single approaches (ARIMA, LSTM, GRU) against hybrid models (ARIMA-LSTM and ARIMA-GRU).The main contribution is to show that hybrid models are effective in working with time series whose beyond the linear component implies the non-linear (fluctuation/residual) component; here, the ARIMA is applied to its linear trend, and the non-linear residuals are interpreted by the neural networks (LSTM or GRU). The hypothesis that the hybridization of statistical and deep learning models can be used to achieve better predictive accuracy and reliability in forecasting the peak load prediction than when based solely on either of the two models was verified by the authors.

Jiao et al.^3^ proposes a sequential strategy to improve the accuracy of one-hour-ahead building energy predictions by addressing the non-stationary and volatile nature of the data. The Complete Ensemble Empirical Mode Decomposition with Adaptive Noise Analysis (CEEMDAN) algorithm is used to decompose original energy data into several Intrinsic Mode Functions (IMFs) to stabilize the signal. The Fuzzy Entropy (FuzzyEn) of each IMF is calculated to measure complexity. Components are then partitioned into high-frequency and low-frequency groups based on their entropy differences. A Random Forest (RF) model predicts the high-frequency components, while a proposed CNN-GRU-Self-attention (CGSA) model extracts spatio-temporal and sequential features from the low-frequency components. Final predictions are derived by superimposing the results of the high and low-frequency sub-models.

Liang et al.^4^ introduces a hybrid framework motivated by a domain knowledge analysis that splits building energy consumption into distinct parts for more reliable long-term forecasting. The building energy consumption is decomposed into three parts: a global part (typical user behavior explained by weather and time), a local part (non-typical behavior/recent trends), and white noise.A Deep Ensemble (DE) static model is trained on meteorological (temperature, wind speed) and time-related (hour, day of week) features to predict the global component. The residuals from the static model are analyzed for autocorrelation and fitted using an Autoregressive (AR) model. The model employs a 24-hr ahead sliding window approach, where final predictions are the sum of the global (DE) and local (AR) forecasted values.

Chen et al.^5^ address the volatility of power load sequences by combining intelligent optimization with deep learning to improve stability and information utilization. The model applies Variational Modal Decomposition (VMD) to decompose complex load sequences into stable IMF components with adjustable amplitudes and frequencies. To optimize VMD parameters and LSTM hyperparameters, the study proposes the Multi-Strategy Optimization Dung Beetle Algorithm (MODBO), an enhanced version of the Dung Beetle Optimizer that mitigates local optima issues using strategies such as reverse learning, spiral search, optimal value bootstrapping, and dynamic weighting. The optimized components are then modeled using LSTM networks to capture long-term temporal dependencies, and the final load forecast is obtained by aggregating the predictions of all components.

Wang et al.^6^ focuses on the specific energy consumption of chiller units in HVAC systems, which accounts for a significant portion of building energy use. The researchers employ wavelet decomposition (using the Daubechies 4 wavelet) to break down the highly random and non-stationary chiller power data into sets of sub-signals. The Pearson correlation coefficient is used during preprocessing to select the most relevant input features (such as frequency of chilled water pumps or condenser pressure) for each decomposed sequence. A multi-layer LSTM neural network serves as the base model to handle the temporal and nonlinear relationships in the sequences. After predicting each subsequence, wavelet reconstruction is used to summarize the results into a final power prediction. El-Kenawy et al.^7^ introduces a hybrid GGO-ARIMA model, which integrates the Auto-Regressive Integrated Moving Average (ARIMA) method with the bio-inspired Greylag Goose Optimization (GGO) algorithm. The model incorporates exogenous variables such as weather, holidays, and academic schedules to capture complex consumption patterns. The GGO-ARIMA model outperformed conventional ARIMA and other optimization-enhanced versions.The GGO algorithm’s adaptive search mechanism effectively balanced global and local exploration to avoid local optima.

### Graph-based spatio-temporal models

Jiang et al.^8^ propose a Spatial–Temporal Fusion Graph Convolutional Network (FSTGRNN) for regional power load forecasting by jointly modeling temporal patterns and spatial dependencies in electricity consumption. Regional loads are represented as graph signals to capture spatial relationships among different units. The model employs a Spatial–Temporal Block (ST-Block), integrating FC-LSTM and GC-GRU to extract temporal and spatial features. A Spatial–Temporal Fusion Block further enhances learning using gating mechanisms and Transformer-based attention to capture both short-term and long-term fluctuations. Experiments on residential and industrial electricity datasets demonstrate that FSTGRNN outperforms several benchmark models in multi-step load forecasting.

Yang et al.^9^suggests a new DoubleExplored Spatio-Temporal Graph Neural Network (DEST-GNN) to multi-site power forecasting in intra-hour Photovoltaic (PV) systems, that is aimed at learning to jointly capture the spatio-temporal interactions between multiple PV stations. This model consists of Sparse Attention-based Adaptive Spatio-Temporal (SAAST) blocks that incorporate a sparse spatial-temporal attention mechanism to screen off low-correlation data and noise and boost the representation of valid spatio-temporal patterns. In addition, DEST-GNN uses an adaptive graph convolution network (GCN), in which an adaptively learned adaptive adjacency matrix is used. This ability to learn in an adaptive manner combined with a temporal convolution network (TCN) enables the neural network to draw the intricate time-varying space dependencies of multi-site PV data without necessarily using fixed graph structures. The model has been proven to be effective based on experimental studying conducted on Alabama and California (NREL) datasets, which contain the idea that DEST-GNN provides the highest prediction performance in comparison to other deep learning baselines in most intra-hour prediction horizons.

Wang et al.^10^ suggest a heterogeneous spatio-temporal graph convolutional network (HST-GCN) to overcome the difficulties with short-term Electric Vehicle (EV) charging demand forecasting, especially considering the heterogeneity of spatial relationships that traditional models fail to consider. The contributions of the model are two-fold; first, the use of heterogeneous graphs, in particular, a geographic one (relying on physical proximity) and a demand one (reliance on Dynamic Time Warping (DTW) distance, reflecting similarities of demands even between spatially distant areas). Second, the adoption of a region specific prediction models that divide charging regions based on graph embedding (DeepWalk) and Point of interest (POI) information whereby specialized prediction layers can be developed instead of a single model that enhances accuracy.

Nguyen et al.^11^present a graph learning based model to enhance fault diagnostics in contemporary power distribution systems that have significant distributed energy resources based on inverter.The traditional fault detection system that tends to use over current relays faces a lot of difficulties in this situation since renewable integration create new fault current properties . To overcome this, authors utilize a graph neural network (GNN) that is based on temporal and spatial recurrence and learns spatial relationships between buses and temporal relationships in voltage measurements by measurement units. This technique facilitates proper fault occurrences identification, fault type and phase identification and fault localization. The proposed model consumes voltage data rather than current signals, unlike traditional scheme that relies on relays, which does not require a lot of relay installations and can be scaled to large systems. The overall tests carried out on the Potsdam microgrid micro- grid and the IEEE 123-node test feeder show that the model is more efficient in generalization and diagnostic performance in comparison with other neural architecture designs.

Wang et al.^12^ presents a high quality deep learning-based framework that is to promote both stability and accuracy of photovoltaic power (PV) prediction. The novelty of the model proposed by the authors consists in the fact that both the geographical and temporal correlation of the distributed PV stations are considered. It creates two-dimensional measurement structure that is anchored on both geographical distances and spatial correlations of PV sites. The model proposed in the study is denoted as ABCGRU and is composed of a bidirectional Convolutional Gated Recurrent Unit (ConvGRU) which is self-attention and encoder-decoder network that is expected to perform well in recording complex spatial-temporal characteristics. The ABCGRU and the benchmark results yielded on the real and benchmark data demonstrated that the method is more abundantly effective in the ultra-short-term PV generation prediction than the eight available predictive models.

### CNN/RNN-based spatio-temporal models

Jalilafer et al.^13^ study proposes a new algorithm dubbed as Spatial Auto Correlation and Convolutional Long Short-Term Memory (SAC-ConvLSTM) of short-term spatial and temporal forecasting of loads in power distribution networks. The methodology deals with the fact that not all power substations in large areas are spatially autocorrelated and this may complicate and reduce accuracy of traditional Convolutional Neural Network (CNN)-Recurrent Neural Network (RNN) models. SAC-ConvLSTM one applies discrete wavelet transform (DWT) to decompose load time-series data into sub-signals. These sub-signals are then evaluated by using spatio-temporal Moran I statistical method to determine the spatial autocorrelation. Sub-signals with a strong spatial dependence are also forecasted with the help of the ConvLSTM algorithm, whereas the agnostic areas are forecasted with the LSTM before reconstructing the load signal that was predicted. Yin et al.^14^ provides Multi-Temporal-Spatial-Scale Temporal Convolutional Network (MTCN) of short-term load forecasting (STLF) of power systems, which is an algorithm that aims to concurrently learn the temporal dynamics and nonlinear relationships in load data. The main input is the implementation of the Multi-Temporal-Spatial-Scale (MTSS) approach throughout the preprocessing, wherein the real world load data is broken down into smaller sections (15 min and 60 min) in both time and space (by type of electricity, i.e., industrial, commercial, other and so on). The reason behind this preprocessing step is to minimize the noise in data and improve the time series properties. The MTCN is designed in the style of a fully-convolution network with residual block and convolution layers and which fits the involved complex patterns.

Wang et al.^15^ establishes a strong spatial-temporal forecasting framework of Photovoltaic (PV) energy known as DAE-GASF-CNN, whose main aim is to forecast despite the contamination of the data. The modeling process is based on downscaling and restructuring of sparse PV data in terms of Pearson correlation coefficient (PCC) and geographical locations correlation in order to enhance efficiency of predictions.The encoded data presented by the denoising autoencoder which helps in recovering historical data by limiting the effect of corruption caused by corrupted samples , enhancing model resiliency. Lastly, Gramian Angular Summation Fields (GASF) is built alongside a Convolutional Neural Network (CNN) (GASF-CNN) to simulate spatial-temporal correlations by converting time series to into 2D image to feed into CNN. Demonstrated by the reports of experimental verification, with datasets of China and the USA, indicates that DAE-GASF-CNN remains active even in high contamination of information (up to 50 percent), even more working than benchmark models and showing a higher training speed and generalization capacity.

Wang et al.^16^ introduces a new ultra-short-term wind farm cluster power forecasting algorithm, which is founded on dynamic spatio-temporal (ST) correlation and hierarchical directed graph construction which is supposed to address the traditional ineffective neural networks challenges of dynamic correlation, dynamic accumulation of prediction bias and dynamic causality among inputs. The major contributions are connected in the following aspects: achieving the description of dynamic ST correlation information by building or designing both power-and-wind-speed spatio-temporal correlation matrices (STCMs), and a new hierarchical directed graph structure. It is an ideal way of modeling the unidirectional causation between wind speed and wind power (and also includes directed edges) and best accomplishes the goal of turning a target node (the number of wind farms), which is N greatly reduced to 1 in the overall process of generating power forecasts which can be very desirable to make the forecasting in large clusters. The proposed method showed outstanding results in improving power forecasting accuracy over other benchmark methods through the use of case studies.

Cavus et al.^17^ proposal ST-CALNet is a hybrid deep learning framework that was developed to enhance short-term load prediction in smart grids, where the renewable integration is intended to be used. This architecture comprises convolutional neural net (CNN) and attentive Long Short-Term Memory (LSTM) network to be in a position to identify spatio-temporal dynamics in complex energy systems. The CNN layers work out spatial correlations of different input variables such as meteorological conditions and the renewable power production as well as the LSTM processes the temporal dependencies within the past demand. The attention mechanism has a special feature of this model, as adapted weighting of the temporal features based on the relevance is used, provides an interpretability degree and the robustness of a model to be higher, assuming variant demand conditions. The suggested approach, its turn, which was trained on the real-world examples such as the data sets of electrical consumption and the data sets of photovoltaic and wind generation, demonstrates more stable improvements than the base deep learning models, achieves greater predictive performance and has more predictable results of errors. The paper contributes positively to the community of intelligent energy management by providing an open, adaptive and data-rich forecasting tool, that could be applied to facilitate the optimal operation of grid and integration of renewable resources.

Feng et al.^18^ proposes new deep learning architecture to forecast accurately the short-term residential electricity consumption. STGNet deals with the issue of spatial and temporal changes in residential load information by using gated fusion mechanism, a method that combines successfully spatial and temporal data. The model has gated temporal convolutional networks (TCNs), coupled with spatially feature layers to progressively learn intricate patterns and correlations among many residential units with time. Besides, the method also exploits a multi-layer design to process long sequences and provide better resistance to prediction, particularly in circumstances of fluctuating consumption patterns. Ample tests on actual-world data have proven that STGNet is better than existing approaches, delivering more accurate load predictions and also being able to better adapt to varying peak loads using an integrated attention model that concentrates on important characteristics. This paper contributes to residential energy management by providing a scalable and interpretable model that can be applied to smart grids applications where short-term load predictions are needed that will be reliable.

### Attention and transformer-based models

Few works have explored advanced deep learning architectures for predicting electrical load, specifically focusing on the transition from traditional models to transformer-based methods. Fan et al.^19^ offered the Parallel Spatio-Temporal Attention-based TCN (PSTA-TCN) architecture that exploits the speed and long memory benefits of the Temporal Convolutional Networks (TCNs) coupled with overcoming the downside of offering equal weights to all features in traditional TCNs. The essence of the contribution is the design of a Parallel Spatio-Temporal Attention (PSTA) mechanism that process on two parallel paths: one of them is the spatial attention that weighs the exogenous features according to their importance, and the other branch is the temporal attention that extracts dependencies between one time step and another inside the attention window. Such attention outputs are further fed to stacked TCN backbones. The presented architecture showed also better prediction accuracy (lowest RMSE and MAE in different prediction problems and window sizes) and an extremely high speed, being trained 14-fold faster than the earlier state-of-the-art DSTP algorithm.

Xiao et al.^20^ proposes a spatial-temporal attention transformer (S2TAT) model that operates concurrently to improve forecasting electric bus charging station loads to the maximum. The analysis deals with the complicated spatial-temporal relationships, which are the products of the interactions between several charging stations and the bus operation flow plans, which are very dynamic. In contrast to traditional time-series and regression-based models, S2TAT jointly trains spatial attention with temporal attention across a single transformer and thus, it can identify both relatively local correlations among neighboring stations and long-term temporal variations in charging demand. The model also brings a synchronous attention mechanism that deals with spatial and temporal strands together to enhance its aptitude to address nonlinear variations and shifting loads trends related to bus entering frequency and route nets. The S2TAT is trained with real-world electric bus data, and its accuracy and adaptability are superior in comparison to the classical deep learning models.

Dong et al.^21^ utilizes a Transformer-based approach designed to predict the energy consumption rate (ECR) of electric buses (EBs) across various driving distances. The model’s architecture consists of two main stages: Transformer Encoder Module used for deep feature extraction, specifically to learn implicit features from historical dynamic time series data, such as driver behavior and bus kinematic characteristics. Second component is Fully Connected (FC) Neural Network. The implicit features extracted by the Transformer are fused with other predetermined environmental factors (e.g., meteorology, road network, and traffic conditions). This fused representation is then processed by an FC network to establish the final mapping for ECR prediction.

Chan et al.^22^ proposes a sparse transformer-based model for electricity load prediction, designed to be up to five times faster than traditional Recurrent Neural Networks (RNNs) during inference. To address the quadratic memory costs of standard Transformers, the model employs a sparse attention pattern (Big Bird). This allows the model to handle much longer input sequences, which is typical for high-frequency electricity data.

SmartFormer model is a graph-nested transformer developed by Saeed et al.^23^ to capture inter-series dependencies in multivariate energy data. The model seamlessly integrates Graph Neural Network (GNN) components, specifically Graph Convolutional Networks (GCN) and Graph Attention Networks (GAT), into the Transformer layers. This allows for an iterative workflow of sequence encoding and graph aggregation.

Moveh et al.^24^ develops a novel transformer-based deep learning framework specifically optimized for forecasting energy consumption across multiple buildings in a smart city. The key innovation is a hierarchical structure that processes building-specific and global features separately. It first builds independent representations for each building and then aggregates them through a global attention layer to capture cross-building correlations. The framework employs adaptive dropout rates and a specialized building dropout technique, which masks random buildings during training to enhance the model’s ability to generalize across different building types and operational pattern.

### Broader spatio-temporal learning applications

Zhang et al.^25^ explores the compound dynamics that affect building Energy Use Intensity (EUI) and Greenhouse Gas Intensity (GHGI) with the help of creating a comprehensive framework that considers spatial and temporal heterogeneity, an important variable that has not received enough attention in former energy prediction studies. The principal methodology relies on the Geographically and Temporally Weighted Regression (GTWR) model that estimates the effects of ten driving forces that concern the building, social, and economic dimensions. The essence of the contribution is the empirical support that integrating the temporal aspect through GTWR is the best in enhancing the reliability of the model compared to the Geographically Weighted Regression (GWR) model. Shao et al.^26^ studies multivariate time series (MTS) datasets across several real-world domains, primarily focusing on systems where sensors are deployed to monitor complex operations. The study introduces BasicTS+, a comprehensive benchmark designed to address inconsistent performance findings and unfair evaluation practices in multivariate time series (MTS) forecasting. By establishing a unified training pipeline and standardized evaluation metrics, the authors enable a reliable comparison of over 30 distinct deep learning solutions. A central contribution of the study is the identification of dataset heterogeneity, categorizing data by its specific temporal patterns and spatial dependencies.

Saffari et al.^27^ in depth examine the application of spatiotemporal deep learning methods to improve the power system analysis and operation.The survey identifies machine learning techniques as discriminative, generative, and reinforcement learning, their mathematical bases, their advantages, and their drawbacks in the dynamic data of the power system. Examples of the relevant areas of application include cyber attack detection, fault detection, and demand response management, and renewable energy forecasting: spatiotemporal learning algorithms can enhance predictive accuracy and resilience of a system. New directions of research in the field of machine learning have also been flagged in the paper that are likely to improve power system analytics by incorporating multi-dimensional aspects of space and time into analytics more reliably. The researchers will find this survey useful since it provides them with a systematic overview of how complex deep learning models may be applied to dealing with the issues of changing the energy balance in the smart grid representative settings. Table 1 summarizes various models.

**Table 1:** Comparative summary of spatio-temporal forecasting model categories in energy-related time series.

Model category	Core modeling strategy	Representative works	Key strengths and limitations
Hybrid decomposition– optimization models	Signal decomposition combined with statistical or deep learning predictors and optimization algorithms	Pierre et al.^2^, Jiao et al.^3^, Liang et al.^4^, Chen et al.^5^, Wang et al.^6^, El-Kenawy et al.Kin^7^	Effectively handle non- stationarity and noise; however, rely on complex preprocessing pipelines and often lack end-to- end trainability
Graph-based spatio-temporal models	Explicit graph construction to encode spatial dependencies, coupled with temporal modeling using RNNs, CNNs, or attention	Jiang et al.^8^, Yang et al.^9^, Wang et al.^12^, Nguyen et al.^11^, Wang et al. ^12^	Strong spatial interpretability and scalability; performance sensitive to graph design and adjacency definition
CNN/RNN-based spatio- temporal models	Convolutional layers for spatial feature extraction combined with recurrent networks for temporal dynamics	Jalilafer et al.^13^, Yin et al.^14^, Wang et al.^15^, Fei Wang et al.^16^, Cavus et al.^17^	Capture local spatial patterns and sequential dependencies effectively; limited in modeling long-range spatial correlations
Attention and transformer- based models	Attention mechanisms or transformer architectures to learn long-range temporal and spatial dependencies	Xiao et al.^20^, Moveh et al.^24^, Dong et al.^21^, Chan et al.^22^, Saeed et al.^23^	Superior long-term dependency modeling and adaptability; higher computational cost and data requirements
Broader spatio-temporal learning applications	General spatio-temporal frameworks applied beyond power systems, including transportation, finance, and climate	Yan Zhang et al.^25^ , Saffari et al.^27^, Shao et al.^26^	Demonstrate cross-domain effectiveness; often require domain-specific adaptation for energy forecasting tasks

### Scope

Despite extensive research on spatio-temporal electricity forecasting, several critical gaps remain that limit the accuracy, adaptability, and applicability of existing models. Many current approaches rely on static spatial graphs or single-scale temporal modeling, which fail to capture the inherently multi-scale and dynamic nature of electricity consumption across regions and time intervals. While models such as FSTGRNN, DEST-GNN, and STGNet attempt to integrate spatial and temporal dependencies, their architectures remain scale-specific and often assume fixed correlations, making them less adaptable to evolving network conditions. Additionally, although recurrent and convolutional models like LSTM, GRU, and TCN effectively capture short-term temporal dependencies, they frequently struggle with long-range patterns, seasonal variations, and temporal drifts in consumption behavior. Furthermore, most models inadequately incorporate exogenous factors such as weather, socio-economic variables, or policy interventions, relying instead on hand-engineered fusion strategies rather than learnable representations. The robustness of these architectures is also limited, as real-world electricity data often contain noise, missing values, and irregular sampling, which many models fail to handle effectively. Another challenge is interpretability; attention mechanisms provide some insight, but the internal reasoning of deep spatio-temporal models often remains opaque, hindering trust and deployment in real-world grids. Finally, existing methods are predominantly domain-specific, focusing separately on wind, PV, or load forecasting, which restricts cross-domain generalization. Addressing these limitations, the present work aims to develop a novel spatial-temporal model that dynamically learns adaptive graph representations, captures multi-scale temporal dependencies, integrates contextual factors, and incorporates attention-driven interpretability, thereby enhancing both the predictive accuracy and practical utility of electricity consumption forecasting in complex energy systems.

In this work, we aim to develop, evaluate, and compare a comprehensive suite of advanced deep learning models for accurate electricity consumption prediction. Our goal is to systematically benchmark foundational sequence models–such as RNNs, LSTMs, and GRUs–against more sophisticated architectures, including attention-based and transformer-based networks, to identify the most effective approach for modeling high-resolution energy data.

Beyond performance benchmarking, we also contribute by proposing a novel spatio-temporal deep learning model that integrates spatial correlations across multiple grid points with temporal dependencies in load patterns. This model is designed to effectively capture the complex, non-linear, and dynamic interactions that characterize electricity demand fluctuations influenced by diverse factors such as weather variations, calendar effects, and regional consumption behavior.

Through this approach, we aim to enhance both the predictive accuracy and interpretability of energy demand forecasting systems, providing valuable insights for grid management, energy optimization, and demand-side planning.

##  Proposed methodology

The approach adopted for this research follows a systematic framework for forecasting electricity consumption. This process encompasses data preparation, model training with hyperparameter optimization, and comprehensive performance analysis. The workflow is structured to ensure a rigorous and reproducible comparison of all implemented deep learning architectures.

### Model

In many real-world applications like sensor networks, traffic forecasting and multivariate time series, it is very important to model sequential data with both temporal dynamics and spatial correlations. Traditional RNN models are able to effectively capture these dependencies but struggle to explicitly model spatial relationships present between the feature. In this work, the term spatial refers to inter-feature relationships within the multivariate input space rather than geographic spatial information. The Spatial-Temporal GRU (ST-GRU) addresses this challenge by explicitly decoupling spatial and temporal modeling into two distinct stages, enabling improved representation learning, efficiency, and predictive accuracy.

In this architecture, electricity consumption is modeled using a hierarchical spatio-temporal framework. At each time step, the spatial module receives a feature vector representing concurrent load-related variables. These features are arranged as structured feature sequences and processed by a GRU to learn inter-feature dependencies within a single time slice. Unlike conventional temporal recurrent models, the proposed spatial GRU is explicitly designed to capture feature-wise correlations rather than temporal evolution.

Conceptually, the spatial GRU treats the multivariate feature set (e.g., load, voltage, weather variables, or other exogenous inputs) as a structured representation at each time point and encodes their interdependencies into a compact latent embedding. This embedding preserves temporal alignment and is subsequently passed to a temporal GRU, which models sequential dependencies across time. This hierarchical design enables efficient compression of spatial correlations while retaining essential information for temporal forecasting.

As time progresses, this process produces a sequence of condensed feature vectors, each representing the spatially encoded information at a particular time step. These vectors are then passed to a temporal GRU cell, which now focuses on modeling the dependencies and dynamics across time. This temporal GRU captures patterns such as daily cycles, trends, and longer-term fluctuations, effectively learning how spatial correlations evolve over the sequence. The output of this temporal GRU is a context vector, which summarizes the complete temporal evolution of the multi-feature data into a single, informative embedding.

**Fig. 1:**
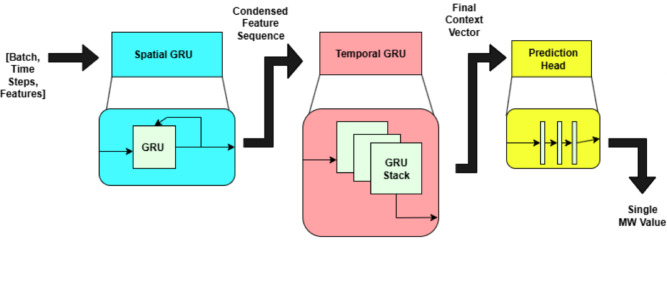
Conceptual architecture of the spatial-temporal GRU model.

Finally, this context vector is passed through a fully connected layer, which maps the learned spatio-temporal representation into the final prediction output, such as electricity demand at the next time step or over a forecasting horizon. By separating spatial and temporal modeling while allowing the two to interact hierarchically, this architecture efficiently captures both feature-wise correlations at each moment and temporal dependencies across sequences, leading to more accurate and robust forecasts. The framework of the proposed ST-GRU is seen in Fig. 1.

While Transformer-based models offer advantages in large-scale parallel processing, GRU architectures are more suitable for deployment in resource-constrained environments such as edge-based smart grid monitoring systems, where lower parameter count and reduced memory footprint are critical. GRUs have fewer parameters and lower computational complexity, making them suitable for lighter models, which implicitly supports their use in resource-limited settings^28,29^.

## Experimental setup

### Datasets

In our study we have employed two publicly available datasets for predicting the electricity consumption. The details of these are provided as follows.

Dataset 1: Wide-format 15-minute interval electricity loads available at Hourly Energy Consumption^30^. This is a complete set of electricity consumption measurements that have been aggregated to 15-minute frequencies and so, there are 96 measures per day in this data set. Such a dataset is arranged in wide-format format, in contrast to the single-index ElectricityLoadDiagrams^31^ dataset the values of which are represented as the load in megawatts (MW): rows are a date and columns are 15-minute intervals of a particular date. The dataset is an extension of several days, which offers high time resolution to be used to learn daily and intra-day consumption patterns. There are a small number of missing or invalid values removed in preprocessing, and the result is a continuous uniform time series. Its regular structure and regular 15 minute granularity make this dataset suitable to train and validate time-series forecasting models prior to estimating them on external test datasets.

Dataset 2: ElectricityLoadDiagrams dataset^31^ is a large-scale time-series collection of records that show electricity consumption from 370 clients over a 4-year time period. The measurements are captured every 15 min, which results in 96 observations per day for each client. The data values are expressed in kilowatts to obtain the energy usage in kilowatt-hours Values should be divided by 4. Each column corresponds to a single client, with zero entries for clients that joined the system after 2011. The dataset contains no missing values. It reflects real-world irregularities, such as time changes due to daylight saving, where March days have 23 h and October days have 25 h. With high temporal resolution and multi-client coverage, it is well-suited for various tasks, and we have decided to make use of this to train and test our model.

### Data preprocessing

Two datasets were utilized in this study, Although both datasets contain 15-minute interval load measurements, their structures differ and therefore required distinct preprocessing pipelines.

Preprocessing of Data set 1 entailed the transformation of the unprocessed wide-form daily format into an equal-time series that could be used to train the model. The initial CSV had the date in the first column, with 15-minute interval columns of loads at each day. A melt operation was used to convert the dataset into three important variables including Date, Time and MW.The Datetime variable was a standardized Date and Time column. Any invalid or missing timestamps were deleted and the MW column was forced to numeric type, deleting non-numeric or missing rows. This step generated an uninterrupted, clean, and uniformly sampled 15 min time series.The data was further chronologically ordered and re-indexed, with continuity in time. The resulting output was the Datetime and MW fields that were first cleaned to create a time series that can be properly ordered and used to make forecasts.Then features and target were scaled using Min-Max, and parameters were fitted using the training split only, to avoid data leakage. Lastly, 96-length sliding windows (24 h of 15-minute time intervals) were created employing $\text {interval\_sin} = \sin \left( \frac{2\pi \cdot \text {interval}}{96}\right)$ to align interval index in the range of 0-95. Sine–cosine encoding preserves cyclic continuity (e.g., hour 23 is close to hour 0), unlike ordinal encoding. Compared to higher-order Fourier terms, first-order sine–cosine encoding was chosen to avoid overparameterization while capturing dominant periodicity. The models were allowed to learn through history sequences to determine how the next step would demand electricity. The process of training and verification of all models in this study adopted dataset 1.

In the case of ElectricityLoadDiagrams Dataset (OPSD style single-index time series) which is referred as Dataset 2 in this article, preprocessing process started with ingestion of the CSV file containing utc-timestamp and load variables. The timestamp column was converted to a UTC - naive DatetimeIndex which is chronologically ordered and searched against duplicates or gaps in order to ensure data integrity at a 15-minute sampling rate. The column that contained the actual electricity load was then chosen and renamed to MW and any observations were cut off. A series of engineered features was also proposed to represent the cyclical temporal dependencies, including the interval of the day, day of the week, month and day of the year all coded using sine and cosine transformations. The dataset was then divided chronologically into training, validation and test infers in 50, 20 and 30 percent respectively. An instance of a MinMax scaler was created on the features and the target with parameters being fit on the training set only to avoid data leakage. An independent MinMaxScaler was fitted exclusively on each dataset’s training split (first 70% of chronological samples), and no scaler parameters were transferred from Dataset 1 to Dataset 2 at any stage.Lastly, 96 (24 h of 15-minute intervals) long sliding windows were built, thus allowing the models to use past sequences of features to predict the demand for electricity in the next step. The data were converted into the 15 minute resolution resulting in an increase in the total number of training sequences (16,500 to 66,385). This helps the model understand a bigger and a more varied range of patterns, and it is easier to converge and be able to capture short-term changes of load demand. Scale Characteristics of these two Datasets as seen in Table 2.

**Table 2:** Scale characteristics of the two datasets.

Dataset	MW range	Mean MW	Train split
DS1	0.0–89.6	42.3	First 70%
DS2	29,158–77,853	55,493	First 70%

### Training and optimization

Adam Optimizer is used with weight decay as a regularization method for model training. Validation loss was used to early stop to prevent overfitting. Sequences Shapes of Batches of data were processed in batches with sequence shapes of (batchsize, seqlen, inputdim).

The process of modeling active forecasts using ST-GRU begins with careful data management and feature engineering to ensure that temporal evolution and correlation among input features are fully exploited.

The development of the Spatial-Temporal GRU (ST-GRU) was aimed at the interaction of features and time dynamics in the future prediction of electricity consumption. The sequence batches in the input layer are shaped (batchsize, 96, 9), with each window = 96 timesteps–24 h–and with 9 features, such as the raw MW value and artificial cyclical encoding of interval of day, day of week, month, and day of year. The Spatial GRU processes the sequences and learns the relation of features in each timestep independent of each other, converting the 9-dimensional feature vectors one after another to a higher-dimensional hidden representation. This gives a cascade of spatial embeddings of shape (batchsize, 96, hidden dim spatial), which intelligently encode the correlations between features without disturbing temporal sequence. The sequence of spatial embeddings is then passed through the Temporal GRU can be used to model short term variability as well as extended temporal dependencies inherent in electricity load data. This component gives out the final hidden state which is then extracted as a parsimonious expression of the original input sequence. This is then subjected to a ReLU activation which is required to adds non-linearity to the mapping, and inhibits negative activations, and then the activation is finally subjected to a fully connected dense layer that produces a scalar which tell us the amount of megawatts to consume in the next 15 min.

The training of the ST-GRU is a supervised learning pipeline which uses sliding window, the input window, and the immediate next-step MW are the target of each window. To ensure that the temporal structure is preserved to prevent the look-ahead bias,Training,validation and test subset(around 50, 20, and 30 per cent, respectively) are created by splitting the dataset. Diagnostic checks are applied to ensure that the windows do not overlap between splits. PyTorch DataLoaders take groups of 64 sequences at once, training batches are shuffled to enhance generalization and validation, and test batches are processed sequentially to preserve reproducibility. The training is done by minimizing mean square error loss and optimized using Adam incorporating weight decay for regularization,the latter will speed up convergence and regularize the model to avoid overfitting. Early stopping loss of validation between epochs and stops training when improvement is not being made within a specified patience period, thus ensuring that the model adapts but not learns by heart the training data. The predictions are then inversely transformed to the original MW scale with scalers trained in the training data. Furthermore, our proposed model and classical recurrent models, Bidirectional recurrent models, attention-augmented RNN, transformer based models, TCN and spatial temporal variants of GRU and LSTM where trained. The hyper parameters common to all models and also model specific hyper parameters are given in Tables 3 and 4.

**Table 3:** Hyperparameter optimization results using Optuna.

Parameter	Search range (Optuna)	Best value found
Hidden size	{64, 128, 256, 512}	512
Number of layers	{1, 2, 3, 4}	2
Dropout rate	0.1 – 0.4	0.2 (fixed)
Learning rate	$2\times 10^{-4}$ – $5\times 10^{-3}$ (log-uniform)	$7.69\times 10^{-4}$
Training epochs	70 – 130 (step 10)	90
Best validation loss (MSE)	Minimised across 20 trials	0.000762
Test RMSE (MW)	–	1.7874
Test accuracy ($\pm 5\%$)	–	82.74%
Test sensitivity	–	92.71%

The Optuna search (20 trials) identified an optimal SpatialTemporalGRU configuration with a learning rate of $7.69\times 10^{-4}$, hidden size of 512, and two recurrent layers.

**Table 4:** Architecture-specific hyperparameter configurations.

Model category	Parameters	Configuration details
Recurrent (RNN/LSTM/GRU)	Hidden Size; Layers; Dropout	256 units; 2 layers; 0.2 dropout
Bidirectional (BiLSTM/BiGRU)	Hidden Size; Directions; Layers	256 units per direction; 2 directions; 2 layers
TCN (Temporal Conv.)	Channels; Kernel; Layers	64 channels per layer; Kernel size = 3; 4 layers; 0.2 dropout
Transformer	$d_{\text {model}}$; $n_{\text {head}}$; $n_{\text {layers}}$; $d_{\text {ff}}$	128 embedding dim; 4 heads; 3 encoder layers; 256 feed-forward; 0.1 dropout
Attention-based	Context Vector Dim; Hidden	256 units; Bidirectional = True; 256 context dim
TS / ST (Hybrid)	Spatial/Temporal Hidden	256 spatial units; 256 temporal units; 2 layers
Spatial temporal GRU	Hidden Size; Layers; Dropout	512 units; 2 layers; 0.2 dropout
BiLSTM-SSA (Optimized)	Search Space (SSA)	Ranges: LR $[10^{-4}, 10^{-2}]$; Hidden [32, 256]; Layers [1, 2]; Weight decay $[10^{-7}, 10^{-4}]$; Dropout [0.1, 0.4]. SSA Config: Population = 5, Iterations = 3.

## Results

Dataset 1 (wide-format 15-minute interval electricity load data) is used to train all the model in this study, and evaluated with Dataset 2 (ElectricityLoadDiagrams, OPSD-style single-index time series). Min-Max normalization was used to scale features and target values, and was trained on the training split of Dataset 1 in order to avoid data leakage. During training, all models were learned exclusively on Dataset 1. Prior to model optimization, input features and target values were normalized using Min-Max scaling, with scaling parameters fitted only on the training split of Dataset 1 to avoid data leakage. Sliding windows of length 96, corresponding to 24 h of 15-minute intervals, were constructed to capture historical load patterns and predict the subsequent time step.

For cross-dataset evaluation, the normalization parameters learned from Dataset 1 were applied unchanged to Dataset 2. This setup reflects a realistic deployment scenario in which a forecasting model trained in one domain is transferred to a related but unseen domain without re-fitting preprocessing parameters. The implications of this assumption on generalization and robustness are analyzed in the “Results” section.

Both data sets were standardized to have a consistent time resolution, feature representation as well as scaling. The input sequences had the format of 96-step sliding windows which represented 24 h of 15 min interval data.

### Other advanced models implemented

Following advanced models were implemented and trained on the dataset. Further these were used for comparing with the proposed framework.

- **Classical recurrent models**: GRU (GRUNet) and LSTM (LSTMNet): Unidirectional recurrent networks that capture temporal dependencies in the sequences. Serve as baseline models due to computational simplicity.
- **Bidirectional recurrent models**: BiGRU (BiGRUNet) and BiLSTM (BiLSTMNet): Integrate forward and backward sequence passes to capture richer temporal context.
- **Attention-augmented RNNs**: GRU Attention and LSTM Attention: Incorporate learnable attention mechanisms on top of bidirectional or unidirectional RNNs to focus on influential timesteps within the input sequences
- **Transformer-based model**: TransformerModel: Models long-range dependencies across sequences without recurrence by making use of self attention.
- **Temporal convolutional networks (TCN)**: Stacked dilated causal 1D convolutions capturing local-to-global temporal patterns efficiently.
- **SpatialTemporal and TemporalSpatial architectures**: SpatialTemporalLSTM: Encode spatial correlations at each timestep before modeling temporal progression. TemporalSpatialGRU/TemporalSpatialLSTM: Model temporal progression per feature first, then aggregate spatial feature interactions.
- **SSA-optimized BiLSTM**: BiLSTM with hyperparameters optimized via Sparrow Search Algorithm (SSA) to improve performance beyond fixed hyperparameter settings.

The hyper parameters used in each of these models are available in Table 4.

### Comparative analysis of models

**Fig. 2:**
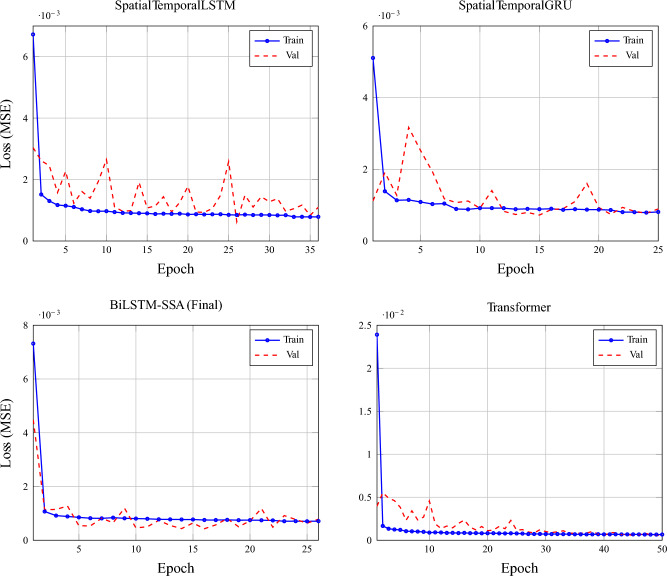
Training vs validation loss comparison for proposed ST-architectures and optimized models.

**Fig. 3:**
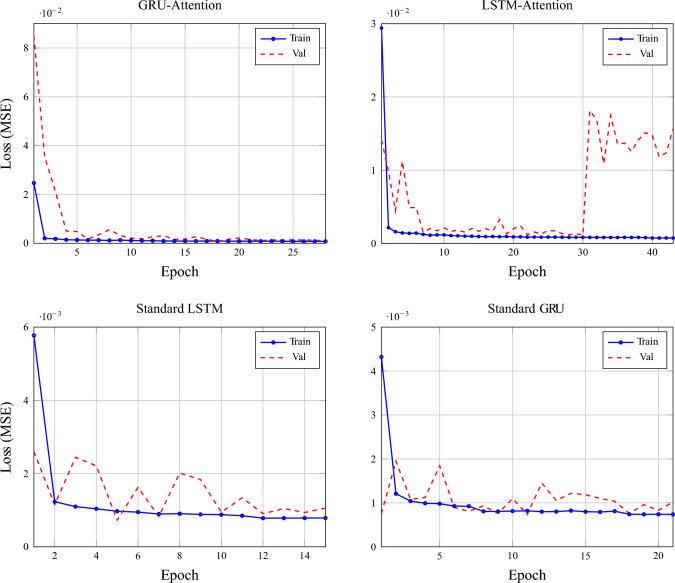
Training vs validation loss comparison for baseline standard models and attention-mechanism augmented versions.

The proposed model is compared against nine baseline and state-of-the-art forecasting models, including both classical and deep learning approaches, to ensure a comprehensive and fair evaluation. These models represent widely adopted and competitive methods reported in recent literature for load and multivariate time-series forecasting. The training and validation loss for each of these models is presented in Figs. 2 and 3. Model comaprsion for predictions is available in Figs. 7 and 8

The quantitative outcomes of the model evaluations are detailed in Tables 6 and 7. Across both datasets, the SpatialTemporalGRU(ST-GRU)achieved least minimum error across all metrics and attained favorable result in for both $\textrm{R}^2$ and accuracy, confirming its superior ability to jointly model spatial and temporal dependencies. Its architecture, which processes feature correlations before temporal evolution, enables richer contextual representation, leading to more stable predictions across varying load patterns.

The SSA-optimized BiLSTM ranked as the second-best overall performer, closely following the SpatialTemporalGRU. The metaheuristic optimization of hyperparameters through the Sparrow Search Algorithm clearly improved convergence and robustness, reducing overfitting and enhancing cross-dataset transferability.

Models such as the Transformer and TCN also demonstrated strong performance, outperforming most classical recurrent architectures. The capability to capture dependencies (Transformer) as well as multiscale temporal patterns (TCN) allowed them to generalize well between the training and testing datasets, indicating their suitability for high-frequency load forecasting. To better understand the contribution of individual components in the proposed architecture, an ablation study was conducted. We evaluate simplified variants of the model by selectively removing or modifying key modules while keeping the training protocol and hyperparameters unchanged. Specifically, the following variants are considered:

- **Temporal-only GRU**: the spatial module is removed, and raw multivariate features are directly fed into the temporal GRU.
- **Spatial -only GRU**: the temporal module is removed, and raw multivariate features are directly fed into the spatial GRU which processes features independently at each timestep
- **Full ST-GRU (proposed)**: the complete hierarchical spatial-temporal architecture.

**Table 5:** Performance comparison of GRU model variants for ablation study.

Variant	RMSE	Accuracy (%)	Sensitivity (%)	Specificity (%)	F1 Score (%)
SpatialOnly_GRU	2.1666	69.35	92.04	94.46	93.40
TemporalOnly_GRU	1.9606	74.73	90.25	97.07	93.57
SpatialTemporal_GRU	1.8008	82.48	93.36	96.40	94.96

The results are shown in Table 5. SpatialTemporalGRU significantly outperforms Spatial- Only GRU, with 17% higher accuracy, 0.37 lower RMSE, and better balance across classification metrics (F1 improves from 93.4% to 95.0%). The temporal component adds substantial value for your spatiotemporal forecasting task.

By contrast, conventional LSTM and GRU networks, as well as their attention-augmented variants, recorded comparatively higher error values and lower accuracy. While attention mechanisms improved interpretability, they did not consistently enhance predictive power when applied on limited-scale data. The SpatialTemporalLSTM showed moderate results, performing better than plain RNNs but lagging behind its GRU-based counterpart, suggesting that GRU cells provide more efficient gradient propagation for this forecasting task.

We found that our experimental results were the same across all repeated runs, and this fact was attributed to Deterministic Inference , All models were run in model eval mode using torch.no grad, which was achieved by disabling stochastic components such as Dropout. We used fixed random seeds and deterministic implementations in the PyTorch environment which eliminated the slight numerical variations typically caused by GPU non-determinism and therefore obtained the same result between different execution cycles on all the metric.

A direct comparison between Dataset 1 (training domain) and Dataset 2 (testing domain) indicates that most models retained their relative performance ranking, demonstrating consistent generalization. However, the absolute values of the metrics slightly increased on Dataset 2 due to differences in temporal dynamics and load distribution across the two datasets. Models with explicit spatial–temporal disentanglement (SpatialTemporalGRU, SpatialTemporalLSTM) and adaptive optimization (BiLSTM SSA) exhibited smaller degradation in accuracy and error, signifying higher robustness to dataset shift.

**Table 6:** Performance comparison of ST-GRU with SOTA models on Dataset1.

Model	MSE	RMSE	MAE	$R^2$	MAPE	Accuracy
SpatialTemporalGRU	3.440899	1.854966	1.297704	0.988025	3.131189	80.681932
TCN	3.671357	1.916078	1.364891	0.987223	3.484675	76.589334
Transformer_Paper	3.587274	1.894010	1.380047	0.987515	3.416942	76.353319
BiLSTM_SSA	3.785608	1.945664	1.409371	0.986825	3.525522	75.218439
GRU	3.933393	1.983278	1.437939	0.986311	3.603918	74.038365
SpatialTemporalLSTM	4.433119	2.105497	1.614144	0.984571	4.102105	65.903385
LSTM_Attention	5.020795	2.240713	1.671338	0.982526	4.213989	65.069800
LSTM	6.122542	2.474377	1.828148	0.978692	4.391959	62.523853
GRU_Attention	5.773315	2.402772	1.903495	0.979907	5.079799	54.760470
BiGRU	5.654719	2.377965	1.899246	0.980320	5.251573	53.510093

**Table 7:** Performance comparison of ST-GRU with SOTA models on Dataset2.

Model	MSE	RMSE	MAE	$R^2$	MAPE	Accuracy
SpatialTemporalGRU	3600626	1897.53	1536.99	0.963525	2.913453	84.343275
TCN	4147679	2036.59	1630.25	0.957983	3.121571	81.713396
Transformer	4073456	2018.28	1626.32	0.958735	3.063385	83.695742
BiLSTM_SSA	3841468	1959.97	1538.69	0.961085	2.873776	83.929252
GRU	4295695	2072.61	1681.46	0.956484	3.203287	80.954905
SpatialTemporalLSTM	4956616	2226.35	1803.16	0.949788	3.470082	77.106139
LSTM_Attention	10422270	3228.35	2526.41	0.894420	4.997114	63.728202
LSTM	8003140	2828.98	2281.10	0.918926	4.221111	65.548250
GRU_Attention	8109544	2847.73	2251.51	0.917848	4.385729	67.217594
BiGRU	3677221	1917.61	1535.01	0.962749	2.854821	83.225411

The performance compared in terms of root mean square error (RMSE), mean absolute error (MAE), mean absolute percentage error (MAPE), $R^2$ and accuracy which provide valuable insight into the models forecasting accuracy and reliability. Here tolerance-based accuracy metric is reported for interpretability. A prediction is considered accurate if

1 $$\begin{aligned} \frac{|y_i - \hat{y}_i|}{y_i} \le \epsilon , \end{aligned}$$

where $\epsilon$ is a fixed tolerance threshold applied uniformly across all models. This metric is supplementary and is reported alongside standard regression measures. Although the model achieved high $\textrm{R}^2$ values, the observed MAPE appears relatively higher. This discrepancy arises due to the scale-dependent nature of MAPE, which disproportionately penalizes prediction errors during low-demand periods. In electricity load forecasting, nighttime or off-peak intervals often exhibit smaller absolute load values, and even moderate absolute deviations result in larger percentage errors. In contrast, $\textrm{R}^2$ reflects the model’s ability to explain overall variance across the full demand range, which remains high due to accurate tracking of peak and mid-range demand patterns. Therefore, the elevated MAPE does not necessarily contradict the strong explanatory performance indicated by $\textrm{R}^2$. We also experimented on various values of $\epsilon$ to understand its impact on GRU and LSTM models. The results are as seen in Table 8.

**Table 8:** Model performance under different error tolerance levels ($\epsilon$).

Model	$\epsilon$ = 1% Acc (%)	$\epsilon$ = 5% Acc (%)	$\epsilon$ = 10% Acc (%)	RMSE
SpatialTemporal_GRU	22.02	82.48	98.03	1.8008
SpatialOnly_GRU	17.16	69.35	90.70	2.1666
SpatialTemporalLSTM	18.70	76.51	97.48	2.0050

SpatialTemporal GRU leads with top $\epsilon$=5% accuracy (82.48%) and lowest RMSE, confirming temporal modeling boosts precision over spatial-only by  13% at key tolerances. All models exceed 90% at $\epsilon$=10%, suitable for practical forecasting.

### Performance visualization

**Fig. 4:**
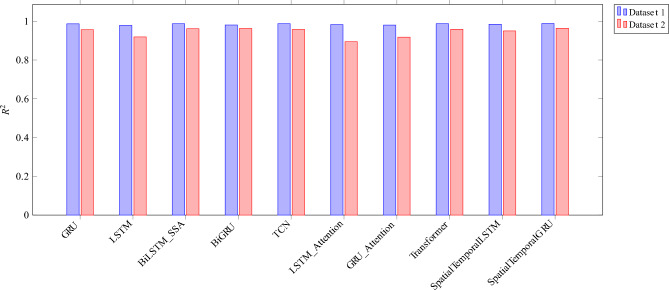
Comparison of $R^2$ on various SOTA models and proposed ST-GRU.

**Fig. 5:**
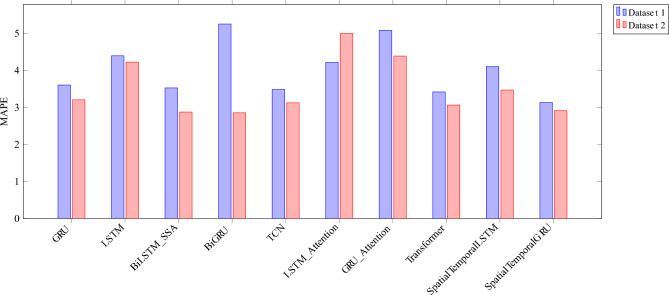
Comparison of MAPE on various SOTA models and proposed ST-GRU.

**Fig. 6:**
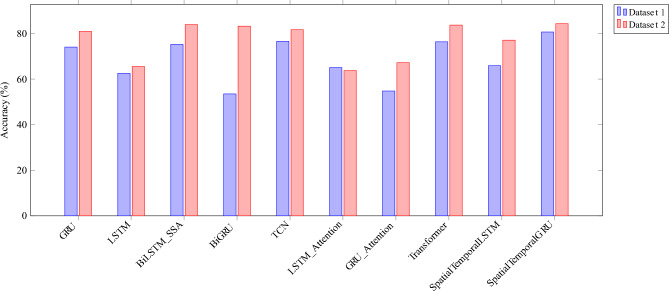
Comparison of accuracy on various SOTA models and proposed ST-GRU.

**Fig. 7:**
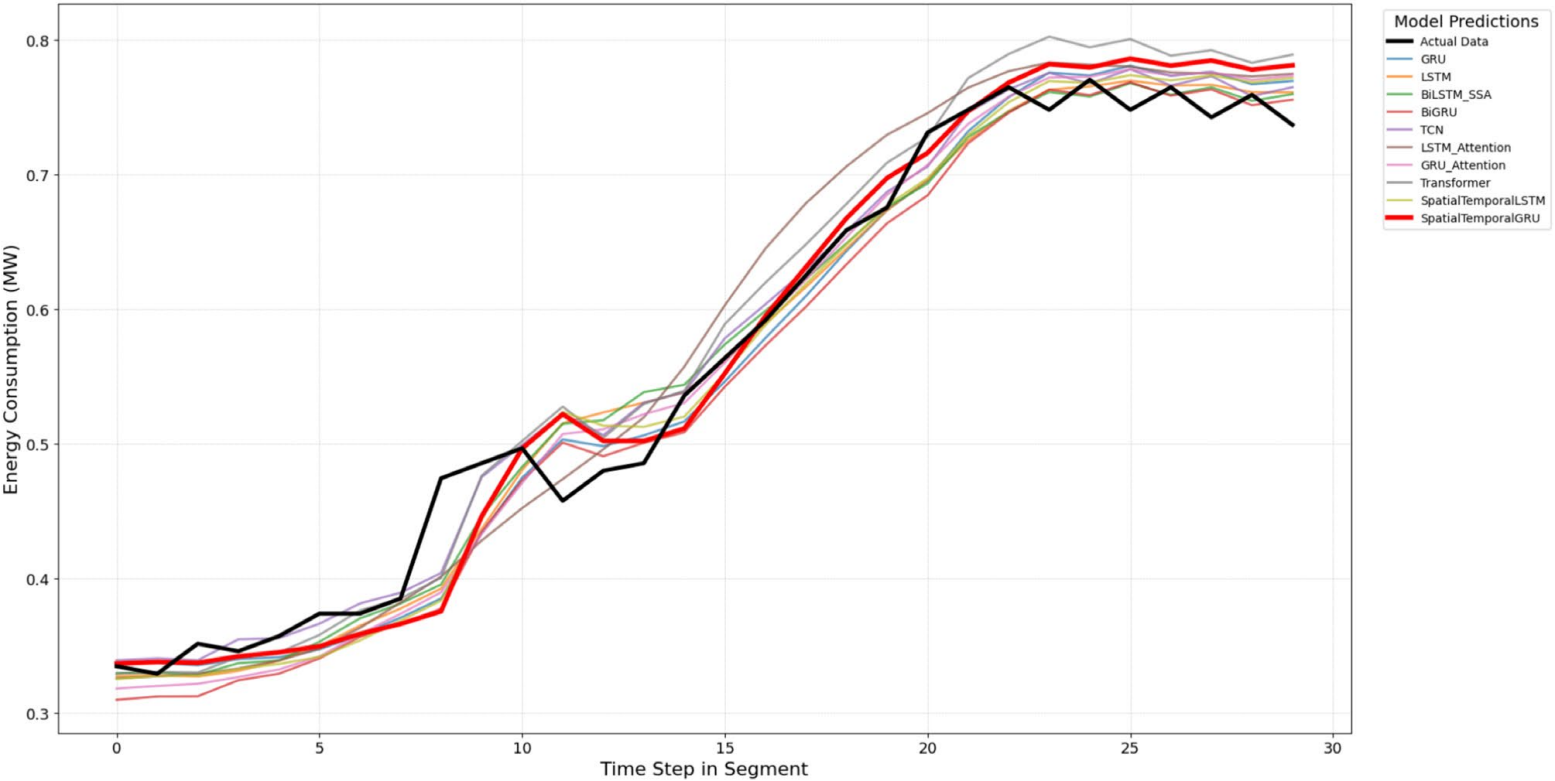
Energy predictions vs. actual for Dataset 1.

**Fig. 8:**
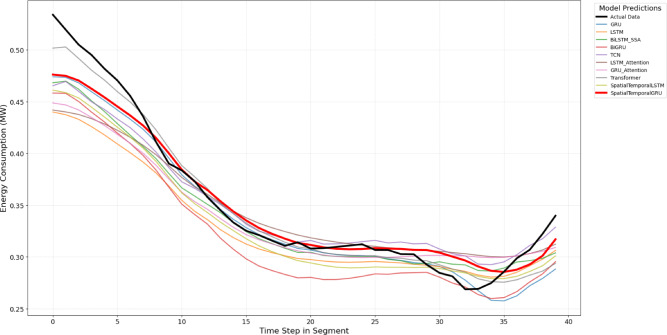
Energy predictions vs. actual for Dataset 2.

Model performance was visualized for qualitative comparison. We compare $R^2$ , MAPE and accuracy of all the models studied in this work and is presented as Figs. 4, 5 and 6. Further, Figs. 7 and 8 presents a series of zoomed-in plots comparing model predictions against the actual data during different critical consumption periods.

Figure 7 is concerned with a test segment from the Dataset 1 in which the energy consumption largely declines, and thereafter, there is a minor recovery. Throughout this shifting trend, the actual data (black line) is most closely adhered to by the SpatialTemporalGRU (red line), which, in effect, measures the decreasing and recovering curves. Other models e.g. LSTM, GRU and Transformer models tend to follow the trend but tend to deviate more with the actual values,particularly in the low turnaround point and the subsequent turnaround. Figure 8 demonstrates a quick growth of the energy consumption taken from Dataset 2. Here as well, SpatialTemporalGRU is capable of following the steep increase in the real data with impressive precision and has a low lag both in the continuous increase and when it accelerates. Competing models such as LSTM and BiLSTM are close but have a tendency to average the fastest gradient which gives a sign that the spatiotemporal-based models are especially good in cases of sharp transitions or highly dynamic situations.we ran several independent evaluation runs on all models.

### Qualitative performance analysis and inferences

####  Window size sensitivity analysis

the experimental setup has been expanded to test shorter and longer windows to demonstrate model robustness. Specifically, the models were evaluated at 48 timesteps (12 h), 96 timesteps (24 h), and 192 timesteps (48 h). The experimental results for the 48-timestep and 192-timestep windows are as seen in Table 9. 

**Table 9:** Performance comparison of Spatial-Temporal LSTM and GRU models under different window configurations.

Model	Window Config	RMSE	Accuracy (%)	Sensitivity (%)
SpatialTemporalLSTM	12h_48ts	1.8892	78.68	93.80
SpatialTemporalGRU	12h_48ts	1.8825	79.54	89.02
SpatialTemporalLSTM	48h_192ts	2.2564	70.01	95.41
SpatialTemporalGRU	48h_192ts	1.8647	80.55	89.93

#### Temporal responsiveness

SpatialTemporalGRU and BiLSTM-SSA show the most stable and responsive predictions, quickly adapts to sudden changes in load pattern such as morning or evening peaks. Sliding window architecture, combines with spatial temporal featuring encoding which allows them to focus on historical cyclic pattern effectively. Transformer capture long range dependencies very well and smooth out short term variations but can underestimate spikes in high frequency data on Dataset2. Classical LSTM and GRU are capable of tracking general trends but lag response to sharp transitions in load series which reflect their limited temporal context capture within fixed length sequence.

#### Handling seasonality and cyclical patterns

Featuring engineering in both dataset benefited all models especially those explicitly leveraging spatial temporal correlation. Spatial temporal architecture successfully understand the cyclic patterns present in the data and make use of it to better align the prediction of peaks peaks and troughs with ground truth. Attention augmented RNNs provide local interpret ability determining to fetch relevant contributing previous time steps to prediction.

#### Robustness to anomalies

To validate the claim that ST-GRU resists temporal anomalies, we conducted stress tests using synthetic data with outliers and missing values. Synthetic corruptions were introduced by adding 5% Gaussian noise spikes and randomly dropping 3% of the values. The degradation in RMSE was evaluated and results are as shown in Table 10.

**Table 10:** Robustness of models under different data corruption scenarios.

Model	Clean RMSE	Gaussian spike (5%)	Random drop (3%)	Combined corruption
SpatialTemporal_GRU	1.8008	4.0969	2.4461 (+ 35.8%)	4.3988
SpatialOnly_GRU	2.1666	8.3836	4.9079 (+ 126.5%)	9.3514

SpatialTemporal GRU shows superior robustness, degrading only 35.8% under random drops vs SpatialOnly GRU’s 126.5%–a 3.5x relative improvement. Under Gaussian spikes, GRU maintains  2.3x better performance (4.10 vs 8.38), confirming temporal modeling enhances resilience to real-world data corruption.

#### MC-dropout uncertainty

Monte Carlo Dropout is a technique used in deep learning to estimate the uncertainty of a model’s predictions without changing the model architecture or retraining it significantly. We performed MC dropout to test the robustness and the results are as shown in Table 11.

**Table 11:** MC-dropout uncertainty evaluation of spatial-temporal models.

Model	Window	MC-RMSE	Mean $\sigma$	95% Cov	Calib r
SpatialTemporalGRU	12h_48ts	2.0187	0.4653	38.00%	0.344
SpatialTemporalLSTM	12h_48ts	2.0004	0.4848	55.00%	0.331
SpatialTemporalGRU	48h_192ts	1.7850	0.5222	53.00%	0.266
SpatialTemporalLSTM	48h_192ts	1.9077	0.4941	50.50%	0.323

SpatialTemporalGRU on 48h-192ts leads with lowest MC-RMSE (1.7850) and best calibration (0.266), though coverage remains moderate across all models (38-55% vs ideal 95%). LSTM provides higher coverage at the cost of slightly worse RMSE.

#### Robustness across datasets

Models trained on dataset 1 are evaluated on Dataset 2 have generalized well since they have retained their performance ranking. Spatial Temporal GRU and BiLSTM SSA have experienced a small amount of degradation in RMSE and MAE showcasing their resilience to changes in underlying load dynamics and unseen temporal changes in contrast to attention based bidirectional RNNs without any hyperparameter optimization which often struggle to provide a stable prediction accuracy has they suffer from large fluctuation reflecting dataset shifts.

#### Interpretability and computational considerations

GRU based model offered faster training and inference speed compared to other models with GRU’s simple gating mechanism which provided simple but efficient gradient propagation over the sequences. Attention mechanism enable insights into important temporal sequence but with a added computation overhead which does not justify its marginal performance gained on mid sized dataset.

#### Practical implications

The evaluation supports the use of Spatial temporal GRU for real world electricity load forecasting due to its accuracy, temporal adaptability and generalization ability. For application where interpret ability of critical timesteps is desired, attention augmented RNNS provide diagonistic insight even if they lag slightly in predictive performance . Models like BiLSTM-SSA offer a robust alternative with optimized hyperparameter, balancing accuracy and stability across datasets.

#### Verification of independent data scaling

To verify that no preprocessing parameters were shared between datasets, we conducted a dedicated scaler robustness experiment on Dataset 2. An independent MinMaxScaler was fitted exclusively on each dataset’s training split (first 70% of chronological samples). No scaler parameters were transferred from Dataset 1 to Dataset 2 at any stage.Two experimental setups were compared as seen in Table 12.

**Setup A: Cross-dataset scaler transfer**: Dataset 1’s MinMaxScaler was applied directly to Dataset 2 without re-fitting. Because Dataset 1 (mean load = 42.3 MW) and Dataset 2 (mean load = 55,492 MW) differ in magnitude by approximately $1.31\times 10^3$, this incorrectly mapped Dataset 2 values to the range [447, 893] rather than [0, 1]. As a result, the model failed to converge, with validation loss remaining above 317,000 across all 20 epochs and a test RMSE of 51,873 MW.

**Setup B: Independent scaler fitting (proposed approach):** A MinMaxScaler was re-fitted on Dataset 2’s training split, correctly normalising values to the [0, 1] interval. Under this configuration, the model converged normally, with validation loss decreasing from 0.0146 to 0.0002 over 20 epochs and achieving a test RMSE of 710 MW. The RMSE reduction of approximately 98.6% between Setup A and Setup B confirms that independent scaler fitting is essential and validates the preprocessing procedure used in this study. The strong generalisation performance on Dataset 2 ($R^2=0.964$, Accuracy = 84.34%) further supports the correctness of the adopted preprocessing strategy.

**Table 12:** Scaler robustness experiment on dataset 2.

Setup	RMSE (MW)	Accuracy (%)	Sensitivity (%)	Specificity (%)	F1 (%)	Best val loss (MSE)
Cross-dataset scaler transfer	51,873.42	0.00	0.00	100.00	0.00	317,341.44
Independent scaler fitting (proposed)	710.32	98.90	98.33	97.79	98.07	0.000198

### Discussion

The outcomes from analyzing the comparative and qualitative studies show important conclusions regarding the performance, generalization, and interpretability of different deep learning architectures developed for the task of short-term electricity load forecasting. The SpatialTemporalGRU model was consistently the most accurate architecture for forecasting electricity consumption across both datasets, The results show that it outperform other models in this application. SpatialTemporalGRU has an unique ability to understand spatial correlations between individual input features and temporal dependencies from timestep to timestep allows it to be the best performing model in relation to MAE, RMSE, and MSE values, showing the largest $\textrm{R}^2$ and accuracy scores for performance evaluation. The model’s performance in generalizing from the characteristics of Dataset 1 (training data) in terms of its prediction capabilities to Dataset 2 (testing dataset) clearly demonstrates the robustness of the model to both temporal and distributional shifts, which is a necessary characteristic of any practice in the utility industry where load behavior changes over time. The SSA-optimized BiLSTM was the next best-performing model, which confirms that, in fact, by optimizing conventional recurrent architectures, one can achieve significantly better performance using any metaheuristic, and the other two models the Transformer and TCN proved to be competitive as well. But it is worth mentioning that their strengths are in modeling long-range dependency, and in the ability to parallel computation. In contrast, the traditional LSTM and GRU architectures showed moderate performance accuracy and generally lagged in rapidly fluctuating periods of the load curve. The model’s ability to understand temporal structure is improved by Attention Layers, but also successfully reduced error on the load prediction models overall, nevertheless, their contribution to the amount of error, was not significant. This could indicate that the improvements gained from using attention maybe more pronounced in larger or noisy datasets or datasets that require modeling high variability, or there are multi-feature dependencies. The Spatial Temporal GRU and BiLSTM SSA showed comparatively similar results when they were applied to two sets of data, pointing to a strong possibly transferable character. Both the Transformer and the TCN models had a slightly bigger degree of deviation in. they were a little more vulnerable to the change in the dataset on their performance metrics, which is their defined temporal dependency. Classical RNNs, in turn, experienced an overwhelming drop in performance, which proves that such models possess low transferability. The clarity and the calculation speed are vital to the working feasibility of forecasting models. GRU architectures were not computationally as expensive as LSTM models, which train faster and made real-time inferences on forecasts with a minimum of loss in accuracy. Attention-based models that were even easier to interpret (in that they provided something about what features were pertinent in their forecasts), increased complexity in terms of computation cost, of their multi-head and alignment blocks. The Transformer needed a lot of attenuation, as well as greater resources. This rendered it more inapplicable to one-off prediction. Taking everything into consideration, the SpatialTemporalGRU had the highest score.

The research focused on the design, development, fine-tuning, and evaluation of a fully layered deep learning model approach to produce model outputs for short-term electricity consumption prediction applications. The aim of this study and research was understand performance of different neural architectures ranging from classical recurrent models (LSTM, GRU types) in addition to using more recent variants enhancing these architectures; including attention-augmented neural networks, bidirectional RNNs, Transformers, Temporal Convolutional Networks (TCN) and Spatial-Temporal hybrids for learning highly complex temporal dependencies contained in high-resolution load data.

This study went farther and considered beyond the predictive accuracy measures to complete the analysis. The research study was specifically targeted on the possibility of the application of the SpatialTemporalGRU architectures in enhancing predictions in electricity consumption prediction. The results of this study are beneficial. Thus we identify a superior course of action towards scalable, intelligence forecasting systems to help in making operational decisions in grid and energy management. The advancement of precise forecasting models holds considerable promise for realizing cost efficiencies, enhancing grid reliability, and supporting data-driven decision-making throughout the energy value chain.

## Conclusion

This research critically evaluated other deep learning-based models of short-term electricity consumption prediction, between classical recurrent models (LSTM, GRU) to new attention, convolutional, transformer, spatial-temporal hybrid on the one hand. architectures. SpatialTemporalGRU model performed well on both the datasets and with lowest forecasting error (MSE, RMSE, MAE) and highest $R^2$ and scores of accuracy. The features of the SpatialTemporalGRU model, namely, the separation of spatial and temporal relationship, made learning easier. of complicated load dynamics whilst being resistant to changes in data distributions. Transformer and TCN-based models on the other hand yielded similar results. They do have faster computation times, but with less accuracy, and susceptible to temporal anomalies in the test data.

The results confirm the advantage of explicitly modeling spatial-temporal correlations, compared to traditional sequence models which only relied on temporal dependencies. The experimental framework investing systematic preprocessing, early stopping, validation-based checkpointing, and hyperparameter searches proved to be effective in stabilizing and generating reproducible models. From an applied perspective, the findings indicate potential for SpatialTemporalGRU based systems to support intelligent energy forecasting and demand-side management in smart grid systems. Moreover, there was clear transferability from Dataset 1 to Dataset 2, suggesting additional generalization potential for new environments and regional datasets. Despite the promising predictive performance demonstrated by the proposed ST-GRU architecture, certain limitations must be acknowledged. First, the model was evaluated on data from a single regional electricity grid, which may limit generalizability across different climatic, demographic, or infrastructure conditions. Second, the current framework does not incorporate exogenous variables such as temperature, humidity, economic activity indicators, or renewable generation penetration, which are known to influence electricity demand patterns. Third, robustness under extreme anomalies (e.g., sudden outages, pandemic-scale demand shifts) was not extensively evaluated. Future work will extend the proposed architecture to multi-region datasets to assess cross-geographical transferability. Incorporating exogenous meteorological and socioeconomic variables may further enhance forecasting accuracy. Additionally, uncertainty-aware modeling and stress-testing under anomalous conditions will be explored to improve reliability for operational deployment in smart grid environments.

In future studies, the predictive robustness can be improved by integrating exogenous factors such as temperature, economic activity, and renewable generation profiles. Exploring an ensemble framework, cross-domain transfer learning, and explainable AI integration will also enhance the interpretation and the upside potential of deploying these methods in operating energy systems.

## Data Availability

Data availability: Data used for the study is taken from publicly available datasets and is appropriately cited. These datasets are available as Dataset1 at https://www.kaggle.com/datasets/robikscube/hourlyenergyconsumption? select=DAYTON_hourly.csv (2023). Accessed: 2025-10-24. Dataset2 available at Trindade, A. Electricity load diagrams 2011-2014. https://archive.ics.uci.edu/ml/datasets/Electricity-LoadDiagrams20112014 (2015). Accessed: 2025-10-24.

## Code availability

The custom code and algorithms developed and used in this study are available from the corresponding author upon request. The code includes implementations of data preprocessing pipelines, model architectures, training procedures, and evaluation metrics necessary to reproduce the findings of this research.
